# Removal of a Large Sarcoma Parotid Gland-Ligature of the External Carotid Artery and Lateral Ligature of the Internal Jugular Vein

**Published:** 1890-04

**Authors:** John A. Wyeth

**Affiliations:** New York, N. Y.; Professor of Surgery in the New York Polyclinic


### Clinical Department

### REMOVAL OF A LARGE SARCOMA PAROTID GLAND —LIGATURE OF THE EXTERNAL CAROTID ARTERY AND LATERAL LIGATURE OF THE INTERNAL JUGULAR VEIN.

JOHN A. WYETH, M. D., PROFESSOR OF SURGERY IN THE NEW YORK POLYCLINIC.

Mrs. C., 56 years old, married for 33 years, three children, all healthy and grown; native England, resident London, Ont., (Canada,) for last 20 years. Family history negative,—25 years ago noticed a small tumor in right parotid region, which attained the size of an almond and was removed by incision 3 years later. Five years after this operation the growth reappeared, but increased very slowly in size for 15 years, when it was about as large as a lemon and projected from the face about 2 inches. In 1888 it began to increase quite rapidly in size, and by the time she came under my care on February 14th, 1890, it measured 5 inches in perpendicular and 4 1-2 inches in transverse measurement It was firmly attached and seemed to develop in the substance of the parotid gland. A diagnosis of sarcoma of the parotid gland was made and operation advised. On Feb'y. 18th it was removed by free dissection—all the integument lying over the tumor was removed with the neoplasm lining; when the operation was completed a wound somewhat elliptical in shape and measuring about 6 x 5 inches.

In the course of the dissection the external carotid artery was exposed and as it passed through the tumor it was tied with catgut ligature. The neoplasm dipped down beneath the range of the lower jaw and in detaching it a sharp venous hemorrhage took place doubtless from a wound of the internal jugular. A forceps was applied and a lateral catgut ligature closed the puncture. By the immediate use of forceps little blood was lost. The large wound was closed by a plastic operation sliding the skin from the cheek backward and from the back of the neck forward to fill the gap. Silk sutures were employed. The patient had a good recovery. The highest temperture was 100 F. She left my house March 6th for Canada.

The facial nerve was necessarily divided as it passed through the tumor. The patient was informed before operating that paralysis of one side of the face would follow.

In one other instance I removed a double sarcoma of both parotid glands, tying both external carotid arteries. Two years later a fair degree of motion had re-appeared in the muscles of one side of the face.

In two other cases I have applied a lateral ligature to the deep jugular vein successfully. The ligation of the external carotid artery has now become a common and accepted operation. It has been done several hundred times with secondary hemorrhage in only very exceptional instances. In my own operations now reaching nearly 20 no hemorrhage has ever occurred.

Sarcomata of the parotid gland are always prone to recur. The time varies from a few months to two or three years. The relief from pain due to pressure and the prolongation of life even for a short period fully justify the small risk of the operation.

